# TRIM27 Negatively Regulates NOD2 by Ubiquitination and Proteasomal Degradation

**DOI:** 10.1371/journal.pone.0041255

**Published:** 2012-07-19

**Authors:** Birte Zurek, Ida Schoultz, Andreas Neerincx, Luisa M. Napolitano, Katharina Birkner, Eveline Bennek, Gernot Sellge, Maria Lerm, Germana Meroni, Johan D. Söderholm, Thomas A. Kufer

**Affiliations:** 1 Institute for Medical Microbiology, Immunology and Hygiene, University of Cologne, Cologne, Germany; 2 Department of Clinical and Experimental Medicine, Faculty of Health Sciences, Linköping University, Linköping, Sweden, and Department of Surgery, Linköping, Sweden; 3 Cluster in Biomedicine (CBM), AREA Science Park, Trieste, Italy; 4 Telethon Institute of Genetics and Medicine, Naples, Italy; 5 Department of Medicine III, University Hospital Aachen, Aachen, Germany; Johns Hopkins School of Medicine, United States of America

## Abstract

NOD2, the nucleotide-binding domain and leucine-rich repeat containing gene family (NLR) member 2 is involved in mediating antimicrobial responses. Dysfunctional NOD2 activity can lead to severe inflammatory disorders, but the regulation of NOD2 is still poorly understood. Recently, proteins of the tripartite motif (TRIM) protein family have emerged as regulators of innate immune responses by acting as E3 ubiquitin ligases. We identified TRIM27 as a new specific binding partner for NOD2. We show that NOD2 physically interacts with TRIM27 via the nucleotide-binding domain, and that NOD2 activation enhances this interaction. Dependent on functional TRIM27, ectopically expressed NOD2 is ubiquitinated with K48-linked ubiquitin chains followed by proteasomal degradation. Accordingly, TRIM27 affects NOD2-mediated pro-inflammatory responses. NOD2 mutations are linked to susceptibility to Crohn's disease. We found that TRIM27 expression is increased in Crohn's disease patients, underscoring a physiological role of TRIM27 in regulating NOD2 signaling. In HeLa cells, TRIM27 is partially localized in the nucleus. We revealed that ectopically expressed NOD2 can shuttle to the nucleus in a Walker A dependent manner, suggesting that NOD2 and TRIM27 might functionally cooperate in the nucleus.

We conclude that TRIM27 negatively regulates NOD2-mediated signaling by degradation of NOD2 and suggest that TRIM27 could be a new target for therapeutic intervention in NOD2-associated diseases.

## Introduction

Innate immune responses are characterized by recognition of invariant structural signatures of pathogens, so called pathogen-associated molecular patterns (PAMPs) as well as the recognition of danger-associated molecular patterns (DAMPs), which generally are compartmentalized host molecules that delocalize upon cell damage. Different types of pattern-recognition receptors (PRRs) show distinct subcellular localizations to recognize extracellular, vesicular or cytosolic PAMPs and DAMPs. NOD2 is a member of the nucleotide-binding domain and leucine-rich repeat containing protein family (NLR), many of which are important cytosolic PRRs [1]–[3]. NOD2 is composed of two N-terminal CARD domains recruiting the downstream signaling adaptor RIP2, a central nucleotide-binding domain (NBD) thought to mediate homooligomerization and a C-terminal leucine-rich repeat domain likely involved in pattern recognition. It is localized to the cytoplasm but is also partially found at the plasma membrane [4]–[7]. NOD2 induces signaling cascades in response to muramyl dipeptide (MDP), a bacterial peptidoglycan fragment, which activates NF-κB and MAP kinases and finally results in the transcription of proinflammatory cytokines, chemokines and antimicrobial peptides [8]–[10].

NOD2 gain-of-function mutations leading to uncontrolled NF-κB activation are found in severe autoinflammatory disorders like early-onset sarcoidosis (EOS) and Blau syndrome (BS) [11], underscoring the need to tightly regulate NOD2 activation. Other mutations in NOD2, predominantly fs1007, are linked with the development of Crohn's disease (CD), a multifactorial inflammatory bowel disease [11]. These mutations are located in the LRR-domain and result in a loss-of-function for MDP sensing. Several proteins like the cell polarity protein Erbin [4], [12], the GTPase-activating protein Centaurin-β1 (CENTB1) [13], and the angio-associated migratory cell protein (AAMP) [14] have been shown to bind NOD2 and negatively regulate NOD2-mediated NF-κB activation. However, the contribution of NOD2 protein turn-over on signaling has not been elucidated.

Tripartite motif-containing (TRIM) proteins are present in all metazoans and over 60 TRIM proteins are encoded in the human genome [15]. They are involved in a broad range of biological processes including cell proliferation, differentiation, development, morphogenesis, and apoptosis and many TRIM proteins are expressed in response to interferons (IFNs) [16], [17]. Members of the TRIM protein superfamily possess a RBCC motif at the N-terminus, which consists of a RING domain and one or two B-Box domains followed by a coiled-coil region. They differ from each other by their C-terminal domain. Most TRIM family members are, however, characterized by a PRY-SPRY (also called B30.2) domain which is suggested to serve as target binding site [18], [19]. RING domains mediate the conjugation of proteins with ubiquitin, SUMO or ISG15 [20], [21] and E3 ubiquitin ligase activity has been observed for several TRIM proteins. Recently, members of the TRIM family have been implicated in regulating antiviral and antimicrobial immune responses. The importance of TRIM proteins in regulating immune homeostasis is highlighted by inherited disorders that are associated with some of these genes: TRIM20 (pyrin) is mutated in the inflammatory disease familial Mediterranean fever (FMF) [22], [23] and TRIM21 (Ro52) is one of the autoantigens detected in Sjögren's syndrome (SS) and systemic lupus erythematosus (SLE), two severe autoimmune disorders [24], [25]. The most prominent example for antiviral activities of TRIM proteins is provided by TRIM5-α which is necessary to restrict retroviral infection in mammals, and in particular in higher primates [26].

TRIM27 (alternatively named RET finger protein (RFP)) is a member of the TRIM superfamily and exhibits the classical PRY-SPRY domain C-terminal of the RBCC motif. TRIM27 was initially identified as a part of the *rfp/ret* transforming gene generated by DNA rearrangements, in which the RING finger is essential for the oncogenic potential [27]. In accordance with the presence of a RING finger, TRIM27 has been shown to confer E3 ubiquitin ligase activity with the enzymes UBE2D1 and D3 [28] and to possess SUMO E3 ligase activity [29]. TRIM27 is highly expressed in mouse spleen and thymus [30] and in cells of the hematopoietic compartment [16]. In contrast to many other TRIM proteins, TRIM27 expression is not altered by type I and II IFNs [16], [17]. TRIM27 exhibits either nuclear or cytosolic localization depending on the cell type [30], [31]. Like other TRIM proteins, TRIM27 can form homooligomers mediated by the coiled-coil domain [31]. Heterooligomerization has been observed between TRIM27 and TRIM19 with the recruitment of the former to promyelocytic leukaemia nuclear bodies (PMLs) [32]. Furthermore, interaction with members of the protein inhibitors of activated STAT (PIAS) family results in targeting of TRIM27 to subnuclear compartments by TRIM27 SUMOylation [33]. A role for TRIM27 in transcriptional repression [34], [35], the negative regulation of NF-κB and IFN-signaling pathways [36], apoptosis [37], and in cell cycle regulation [38] has been reported, suggesting that TRIM27 is involved in the control of multiple cellular processes.

Here we show that TRIM27 regulates innate immune responses by physical interaction with NOD2. We find that TRIM27 mediates K48-linked ubiquitination and subsequent proteasomal degradation of NOD2. Functionally, TRIM27 negatively influenced NOD2-mediated NF-κB activation. TRIM27 expression was enhanced in Crohn's patients, indicating a physiological role of TRIM27 in controlling NOD2 activity. Of note, ectopically expressed NOD2 colocalized with TRIM27 to the nucleus of HeLa cells, suggesting a new role for NOD2 in nuclear processes.

## Results

### TRIM27 is a new interaction partner of NOD2

**Figure 1:**
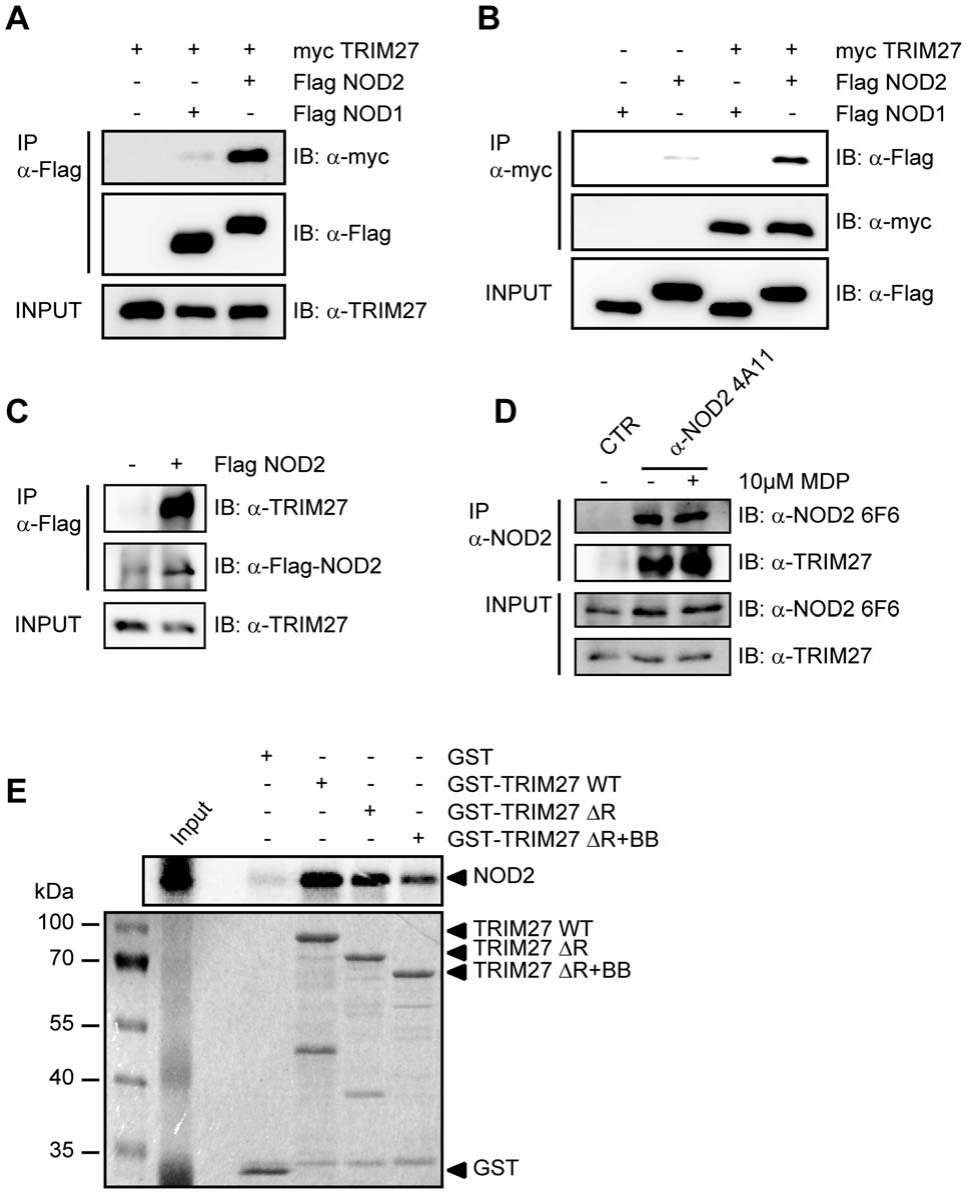
NOD2 physically interacts with TRIM27 via the NBD domain. A–C) Lysates of HEK293T cells expressing the indicated proteins were subjected to immunoprecipitation using anti-Flag (A and C) or anti-myc beads (B). Immunoblots of immunoprecipitates (IP) and total lysates (Input) were performed using the indicated antibodies. D) Immunoprecipitation of endogenous NOD2 from SW480 cells using the NOD2- specific monoclonal antibody 6F6. Cells were treated with 10 µM MDP for 3 h as indicated. Immunoblots of immunoprecipitates (IP) and total lysates (Input) were performed using the indicated antibodies. E) Protein-protein binding assays using *in vitro* transcribed and translated [35S]-methionine-labeled NOD2 and recombinant GST or GST-TRIM27 (WT, ΔRING or ΔRING+B-Box) bound to glutathione-Sepharose beads. The coomassie-stained gel (bottom) and the autoradiograph (top) of co-precipitated NOD2 are shown.

Members of the TRIM protein family are emerging as important new regulators of innate immune responses. We were interested in whether TRIM proteins also contribute to NOD2 signaling. To search for TRIM proteins that physically interact with human NOD2, we conducted a yeast two-hybrid screen with a panel of human TRIM proteins as bait and NOD2 as prey. This identified interactions of NOD2 with TRIM8, TRIM27, and TRIM50 (Figure S1A). Of note, we observed homointeractions for all TRIM proteins indicating a correct folding [39]. Furthermore, TRIM27 formed heterointeractions with TRIM8 and TRIM18 (Figure S1A). Next, we verified interactions of NOD2 with TRIM8, TRIM27, and TRIM50. To this end, co-immunoprecipitations in human embryonic kidney (HEK293T) cells expressing Flag-NOD2 and myc-tagged TRIM proteins were performed. We observed interaction of NOD2 with TRIM27 (Figure 1A), but no robust interaction with the other TRIM proteins tested could be detected (data not shown). Importantly, NOD2 had a much higher binding affinity for TRIM27 than NOD1 (Figure 1A, 1B), which was confirmed by reciprocal co-immunoprecipitations (Figure 1B), suggesting specificity of the interaction.

To establish this interaction for endogenous TRIM27, we transfected HEK293T cells, which strongly express TRIM27 (Figure S2A), with Flag-NOD2 and immunoprecipitated NOD2. This revealed that also endogenous TRIM27 co-precipitated with NOD2 but not with the matrix alone (Figure 1C). Moreover, endogenous TRIM27 also bound endogenous NOD2 precipitated with the NOD2 specific antibody 6F6 (Figure S1B) from the colon cell line SW480 (Figure 1D). NOD2 is activated by MDP, a bacterial peptidoglycan fragment. To explore whether activation of NOD2 has an influence on NOD2-TRIM27 complex formation, we treated Nod2-expressing HEK293T cells with MDP prior to lysis. This revealed that NOD2 activation slightly increased its interaction with TRIM27 (Figure 1D).

To obtain additional *in vitro* evidence for the NOD2-TRIM27 interaction and to elucidate the necessary TRIM27 domain for interaction, we next performed protein-protein interaction assays. To this end, *in vitro*-transcribed and -translated NOD2 was incubated with recombinantly expressed, GST-tagged TRIM27 protein bound to glutathione-Sepharose. NOD2 strongly bound to TRIM27 WT and TRIM27 ΔRING and to a weaker extent to TRIM27 ΔRING+B-Box. Virtually no binding was observed to GST alone, which was used as control (Figure 1E). As reticulocyte lysates only contain a limited repertoire of endogenous proteins, this finding strongly suggest that the NOD2-TRIM27 interaction is direct. Collectively, these findings established that TRIM27 physically interacts with NOD2 in human cells.

### TRIM27 PRY-SPRY and NOD2 NBD domains are sufficient for interaction

**Figure 2:**
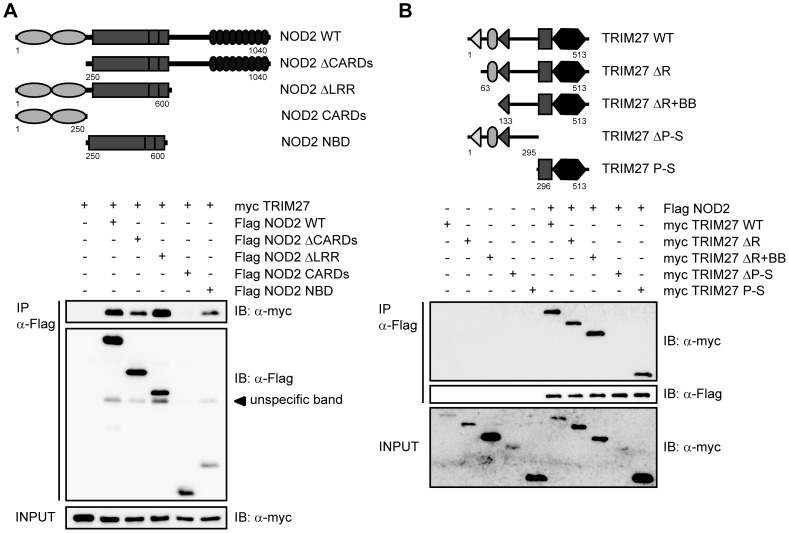
Mapping of the interaction domains in NOD2 and TRIM27. The depicted NOD2 (A) or TRIM27 (B) deletion constructs (upper panels) were used for the domain mapping. Lysates were subjected to immunoprecipitation using anti-Flag beads and immunoblotted with the indicated antibodies.

In order to elucidate the protein domains that mediate NOD2-TRIM27 interaction in more detail, we performed a series of co-immunoprecipitation experiments with NOD2 deletion mutants lacking the two CARD domains (ΔCARDs) or the LRRs (ΔLRR), or comprising only the CARD (CARDs) or NBD (NBD) domains (depicted in Figure 2A, upper panel). We found that NOD2 binding to TRIM27 was independent of the CARD domains and the LRRs. Instead, NOD2 NBD was sufficient to mediate the interaction (Figure 2A, lower panel). Of note, TRIM27 binding to NOD2 ΔLRR appeared to be stronger than to NOD2 WT. Next, we determined which domains in TRIM27 are needed for NOD2 binding. In agreement with our in vitro data (Figure 1E), deletion of the RING or both RING and B-Box domains did not abolish the interaction with NOD2. In contrast, we did not observe NOD2 binding to TRIM27 lacking the PRY-SPRY domain (TRIM27 ΔPRY-SPRY) (Figure 2B, lower panel). However, the expression of this construct was weak and we failed to obtain a construct of this domain that expressed well in human cells. We thus cannot formally exclude that NOD2 weakly binds to this domain. However, we observed strong interaction of NOD2 with the TRIM27 PRY-SPRY domain alone (Figure 2B, lower panel), showing that the PRY-SPRY domain is sufficient for NOD2 binding.

### TRIM27 contributes to NOD2 ubiquitination

**Figure 3:**
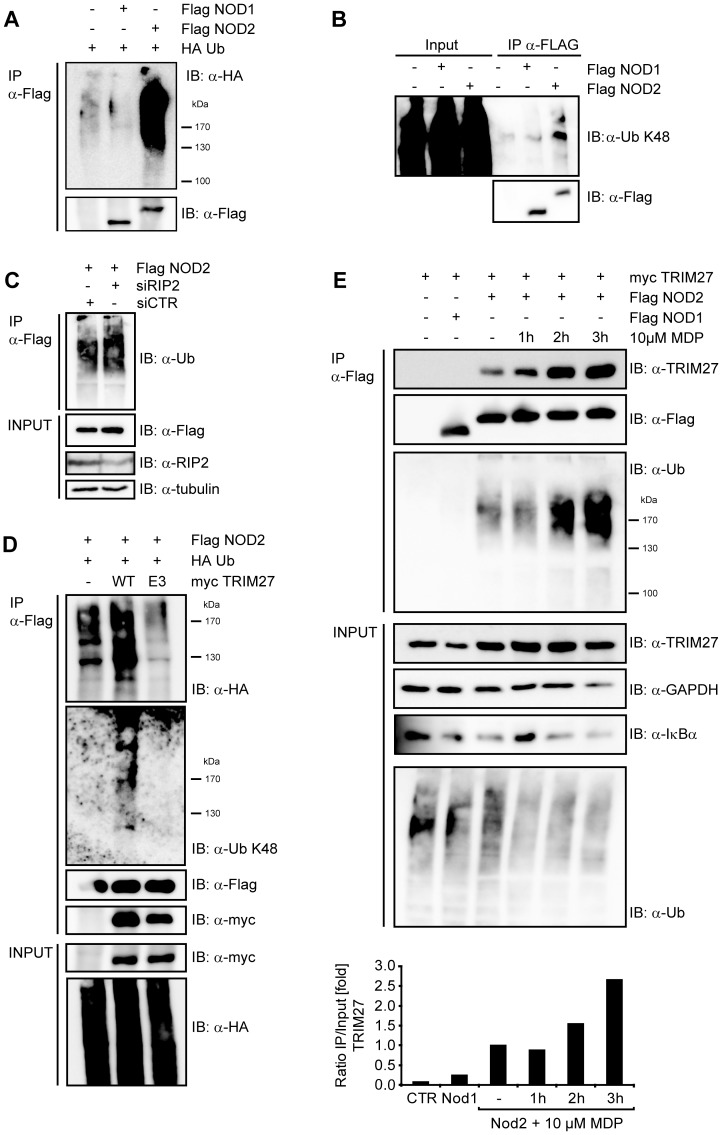
NOD2 is ubiquitinated. A) To determine NOD1 and NOD2 ubiquitination, denatured lysates of HEK293T cells expressing the indicated proteins were subjected to immunoprecipitation. Immunoprecipitates were immunoblotted using the indicated antibodies B) To determine the type of ubiquitin-linkage, lysates of HEK293T cells expressing the NOD1 or NOD2 were subjected to immunoprecipitation. Endogenous ubiquitin was revealed using a K48-link specific antibody. C) HEK293T cells were transfected for 72 h with siCTR, siTRIM27-1 or -3 after expression of Flag-NOD2 and HA-Ubiquitin. Immunoblots of immunoprecipitates (IP) and total lysates (Input) were performed using the indicated antibodies. D) TRIM27 and a E3 mutant of TRIM27 were expressed together with NOD2 and HA-ubiquitin in HEK293T cells. Immunoprecipitates were probed with the indicated antibodies. E) HEK293T cells expressing the indicated proteins were stimulated with 10 µM MDP for the indicated time. Lysates were subjected to immunoprecipitation as described in (C). Densitometric analysis of the TRIM27 signal in the immunoprecipitation normalized to the input signal is shown (bottom). Representative data of at least three independent experiments are shown.

To investigate if TRIM27 might be involved in ubiquitination of NOD2, we first determined basal NOD2 ubiquitination status in human cells. During expression in HEK293T cells, NOD2 but not NOD1 was poly-ubiquitinated at steady-state conditions as revealed by detection of conjugated HA-ubiquitin in immunoprecipitations of denatured lysates (Figure 3A). Using linkage-specific anti-ubiquitin antibodies we showed that the NOD2 ubiquitin chains reacted with an anti-K48-antibody (Figure 3B). Importantly, RIP2, a known NOD2-interacting protein that is ubiquitinated upon NOD2 activation, did not significantly contribute to the ubiquitin signal detected in the NOD2 precipitate, as shown by siRNA-mediated depletion of RIP2 (Figure 3C). Recently, TRIM27 was shown to interact with a subset of E2 enzymes and to possess E3 ubiquitin ligase activity *in vitro* [28]. To investigate if TRIM27 contributes to the ubiquitination of NOD2, HEK293T cells were co-transfected with Flag-NOD2, myc-TRIM27 WT and HA-ubiquitin. The ubiquitin signal in NOD2 precipitates, detected either by anti-HA or the K48-linkage- specific antibody, was increased upon TRIM27 overexpression (Figure 3D). In contrast, overexpression of a TRIM27 E3 ligase-deficient mutant (called ‘TRIM27 E3’ here), in which the two conserved catalytic cysteine residues within the TRIM27 RING domain (C16 and C31) were mutated to alanine, significantly reduced NOD2 ubiquitination compared to TRIM27 WT (Figure 3D). Of note, TRIM27 E3 still bound NOD2 to the same extent as TRIM27 WT, showing that this mutation did not affect binding affinity (Figure 3D).

Since NOD2 activation by MDP seemed to increase the binding affinity of the endogenous protein for TRIM27 (Figure 1D) we went on to explore in more detail how activation of NOD2 influences NOD2-TRIM27 complex formation and NOD2 ubiquitination. To this end, we performed co-immunoprecipitations in HEK293T cells expressing Flag-NOD2 and myc-TRIM27 following stimulation with 10 µM MDP at different time points. MDP induced NOD2 activation as monitored by IκBα degradation (Figure 3E). Moreover, binding of TRIM27 to NOD2 was enhanced over two-fold at 3 h after MDP stimulation compared to untreated controls and accordingly we observed a stronger ubiquitination of NOD2 after MDP treatment (Figure 3E).

Taken together, these data showed that NOD2 but not NOD1 is ubiquitinated and revealed a role for TRIM27 in this process, which was dependent on activation of NOD2.

### TRIM27 contributes to proteasomal degradation of NOD2

**Figure 4:**
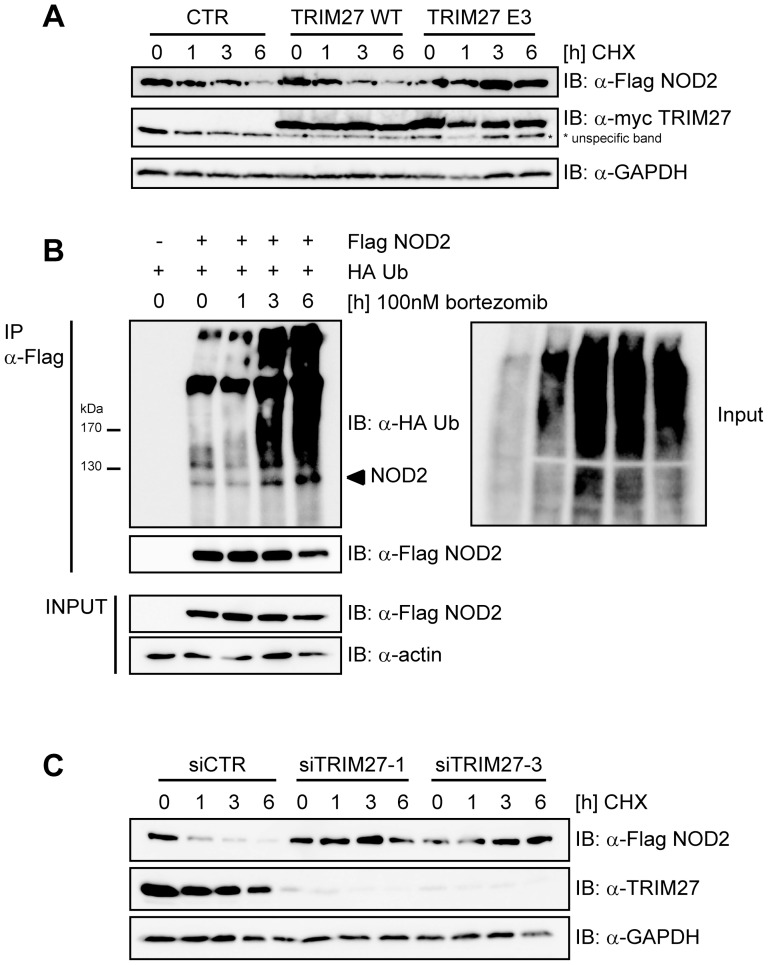
TRIM27 contributes to proteasomal degradation of NOD2. A) HEK293T cells transfected with low amounts of Flag-NOD2 and myc-TRIM27, E3 or CTR as indicated were treated with 30 µg/ml cycloheximid (CHX) and immunoblots of total cell lysates (top) were performed using the indicated antibodies. GAPDH served as loading control. B) HEK293T cells expressing the indicated proteins were treated with 100 nM bortezomib. Lysates were subjected to immunoprecipitation as described in Figure 1A. Actin served as loading control. C) HEK293T cells transfected for 48 h with siCTR, siTRIM27-1 or -3 and subsequently with Flag-NOD2 were treated with 30 µg/ml CHX, as indicated. Immunoblots of total cell lysates were performed using the indicated antibodies. GAPDH served as loading control. Representative data of at least three independent experiments are shown (see also Fig. S3B).

K48-linked ubiquitination of proteins usually targets them for degradation by the 26S proteasome [40]. To investigate if NOD2 is subjected to proteasomal degradation, HEK293T cells expressing small amounts of Flag-NOD2 were treated with cycloheximide (CHX) to block protein neosynthesis and changes in NOD2 protein levels were followed by immunoblot analysis. This showed that NOD2 was readily degraded in a time-dependent manner (Figure 4A, upper panel), whereas NOD1 was not subjected to rapid protein turn-over (Figure S3A). TRIM27 WT overexpression only very slightly influenced the kinetic of NOD2 degradation (Figures 4A, upper panel, and S3B). However, overexpression of TRIM27 E3 strongly inhibited NOD2 degradation. This indicates that TRIM27 E3 acts as dominant negative over TRIM27 WT and endogenous TRIM27, which is expressed in these cells. To determine whether NOD2 degradation occurred via the 26S proteasome, we used the proteasome inhibitor bortezomib (MG-341). NOD2 degradation was inhibited in HEK293T cells expressing Flag-NOD2 treated with 100 nM bortezomib and poly-ubiquitinated NOD2 was strongly accumulated as early as 3 h after bortezomib treatment (Figure 4B).

To elucidate the contribution of endogenous TRIM27 on NOD2 degradation, TRIM27 expression was silenced in HEK293T using two different TRIM27-specific siRNA duplexes. Both siRNA duplexes reduced TRIM27 levels nearly to the detection limit as shown by immunoblot (Figure 4C). In cells transfected with non-targeting control siRNA NOD2 was readily degraded after CHX treatment as observed before (Figure 4C). In contrast, in cells treated with either of the two TRIM27-specific siRNA duplexes, NOD2 degradation was effectively inhibited.

Taken together, these data revealed that K48-linked ubiquitination occurred on NOD2 and that this targeted NOD2 for 26S proteasomal degradation in a TRIM27-dependent manner.

### TRIM27 co-localized with ectopically expressed NOD2 in the nucleus

**Figure 5:**
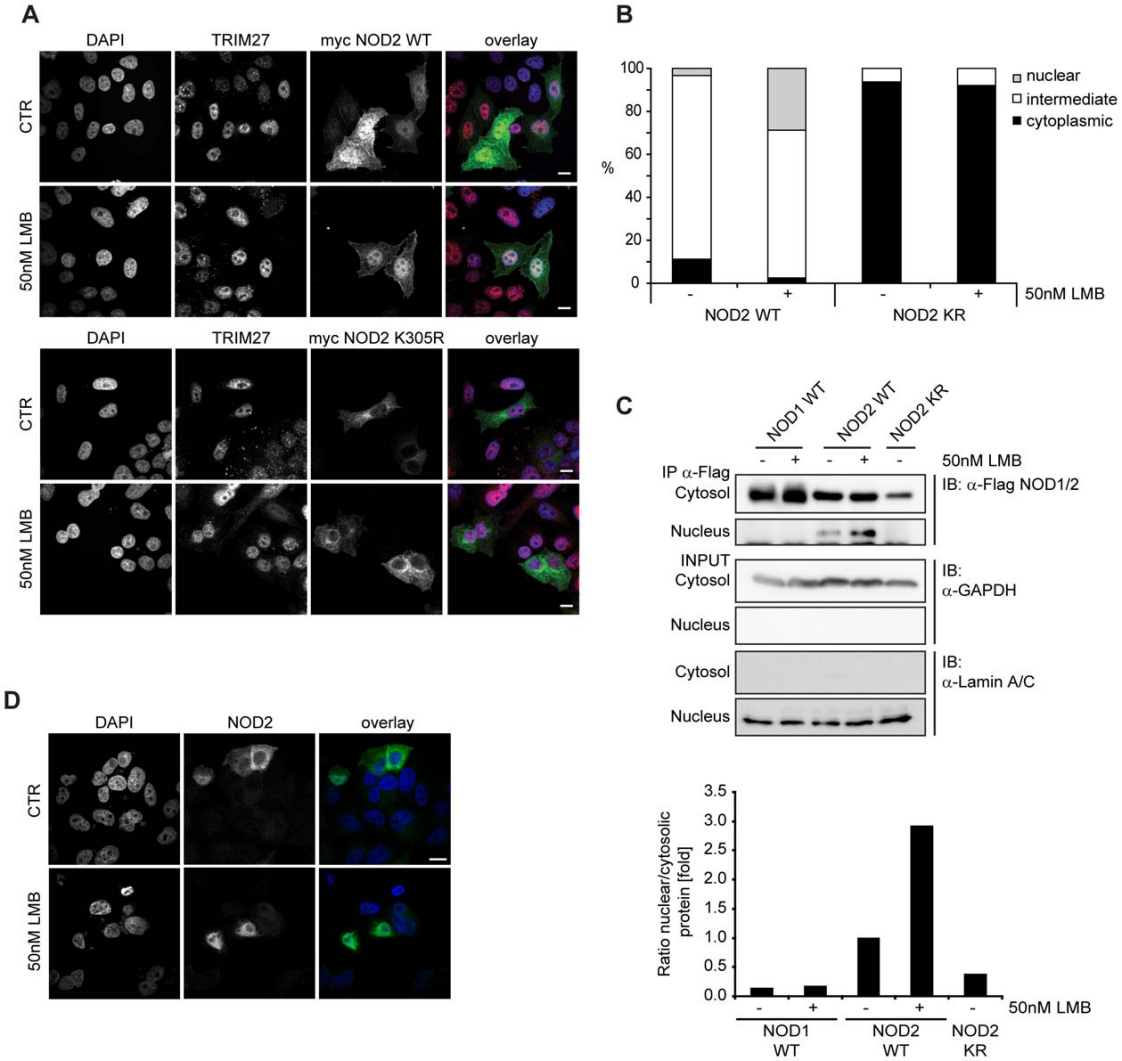
NOD2 WT localizes to the nucleus whereas NOD2 K305R or NOD1 do not. A) Indirect immunofluorescence micrographs of HeLa cells grown on coverslips and expressing myc-NOD2 WT (top) or myc-NOD2 K305R (bottom) were treated with 50 nM leptomycine B (LMB) for 4 h or left untreated. Images with signals for DAPI, TRIM27, myc-NOD2 and an overlay (blue: DAPI, red: TRIM27, green: NOD2) are shown. B) Quantification of NOD2 subcellular localization in HeLa cells expressing Flag-NOD2 WT or K305R, as indicated (n = 100). Data is representative of two independent experiments. C) Cellular fractions of HEK293T cells transfected with Flag-NOD1, -NOD2 WT or -NOD2 K305R, as indicated, and treated as described in A were generated. Immunoblots of precipitated protein and total lysates are shown. For densitometric analysis, cytosolic and nuclear signals were normalized to GAPDH and Lamin A/C, respectively. The ratio of nuclear to cytosolic protein is shown in fold. Representative data of at least three independent experiments are shown. D) Indirect immunofluorescence micrographs of HeLa cells expressing untagged-NOD2 WT treated with 50 nM leptomycine B (LMB) for 4 h or left untreated. Bars, 10 µm.

In HeLa cells, TRIM27 has been shown to localize to the nucleus [31], whereas in HEK293T cells TRIM27 is also found at distinct sub-cellular structures (Figure S2B, S2C). NOD2 has been shown to be a cytoplasmic protein that partially localizes to the plasma membrane [4]–[6], whereas other NLR proteins such as NLRC5 and CIITA also show nuclear localization [41]–[45]. We asked if NOD2 might also be able to shuttle to the nucleus and co-localize in this compartment with TRIM27 as previous experiments indicated that transiently overexpressed NOD2 is sometimes found in the nucleus [4]. To investigate this in more detail, we performed indirect immunofluorescence analysis in HeLa cells transfected with myc-NOD2 WT and blocked nuclear export using the specific nuclear export inhibitor leptomycin B (LMB). In the untreated samples, NOD2 WT mainly localized to the cytosol and was only partially found in the nucleus in less than 5% of the cells (Figure 5A, 5B). After LMB treatment, however, NOD2 WT was found in the nucleus in about 30% of the cells (Figure 5A, 5B). In contrast, NOD1 and a NOD2 Walker A mutant (K305R), which is unable to induce NF-κB activation [8], did not localize to the nucleus at all, even after LMB treatment (Figure 5A and Figure S2D). To substantiate these results, we prepared cytosolic and nuclear fractions from HEK293T cells expressing either Flag-NOD1, -NOD2 or -NOD2 K305R. Immunoprecipitation of the Flag-tagged proteins from the cytosolic and nuclear fractions revealed that a small portion of NOD2 was present in the nucleus without LMB treatment (Figure 5C). NOD2 nuclear localization was enhanced by about three-fold upon LMB treatment, as determined by normalized densitometric analysis (Figure 5C), which is in line with the data obtained in the cell-biological analysis. In contrast, NOD1 and NOD2 K305R were again found exclusively in the cytoplasm even after LMB treatment (Figure 5C). Importantly, also untagged NOD2 showed a nuclear enrichment after LMB treatment (Figure 5D), showing that nuclear localization of NOD2 is not an artifact of the epitope tag used above. Of note, the ability of NOD2 to trigger NF-κB activation upon MDP stimulation was unaffected when NOD2 was targeted to the nucleus by fusing it to a SV40 nuclear localization signal, showing that nuclear localization of NOD2 is not detrimental to its established function in bacterial sensing (data not shown).

Conclusively, our data show that at least ectopically expressed NOD2 is able to shuttle to the nucleus in a Walker A-dependent manner, which is reminiscent of the behavior of other NLRs, such as NLRC5 and CIITA [41]–[45].

### TRIM27 negatively influences NOD2 signaling

**Figure 6:**
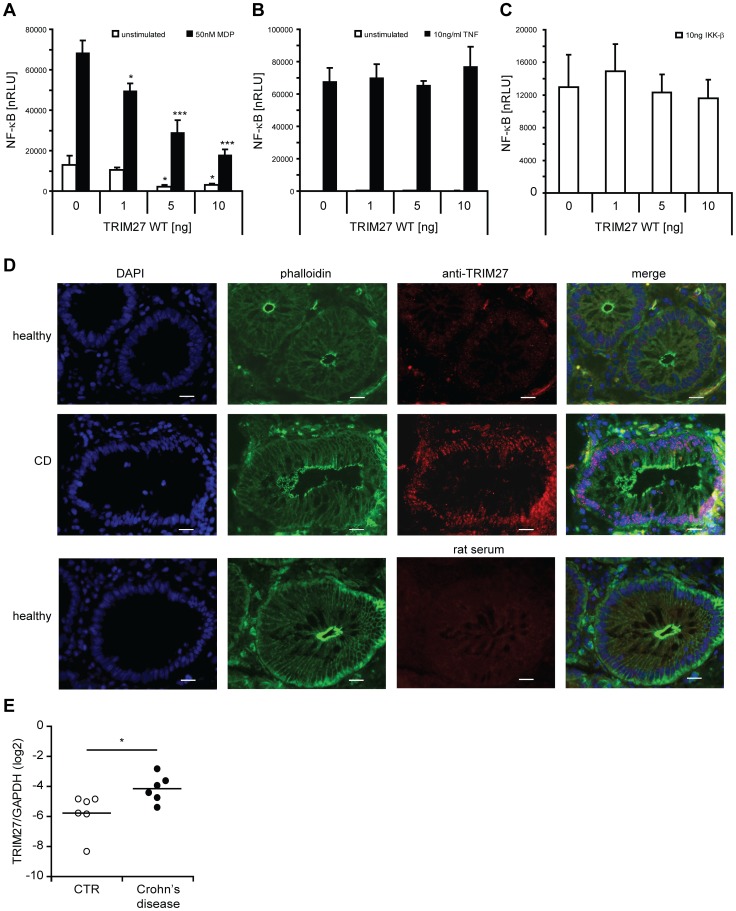
TRIM27 negatively regulates NOD2 signaling. A–C) NF-κB luciferase assays in HEK293T cells to determine the influence of TRIM27 overexpression on MDP-induced NOD2-mediated (A), TNF- (B), or IKK-β-induced (C) NF-κB activation. Normalized luciferase activity (nRLU) of unstimulated (white bars) and stimulated (black bars) samples is shown. Values are given as mean+SD (n = 3). *, P<0.05; ***, P<0.005. *D.* Sections from human colon derived from healthy and active Crohn's disease patients. Staining with DAPI (blue), phalloidin-FITC (green) and with α-TRIM27 antibody (red) is shown. Staining with a rat serum is shown as control in the lower panel (rat serum). Slides are representative for 3 control and 4 Crohn's disease patients. Scale bar = 20 µm. *E.* qRT-PCR of TRIM27 mRNA expression in colonic biopsies derived from patients with Crohn's disease and controls (CTR) with no evidence of mucosal inflammation. Each symbol represents one patient. TRIM27 mRNA expression normalized to GAPDH is shown. *, P<0.05 (n = 6).

Finally, we wanted to determine the influence of TRIM27 on NOD2-mediated signaling. In HEK293T cells, overexpression of TRIM27 significantly reduced MDP-induced NOD2-mediated NF-κB activation in a dose-dependent manner (Figure 6A). This effect of TRIM27 on NOD2-mediated signaling cannot be explained by inhibition of RIP2-NOD2 complex formation as NOD2 was still able to bind RIP2 in the presence of TRIM27 (Figure S4A). Importantly, TRIM27 overexpression had no significant influence on TNF-induced NF-κB activation (Figure 6B). Moreover, NF-κB activation induced by overexpression of IKK-β was also not significantly influenced by TRIM27 (Figure 6C), suggesting that TRIM27 acts upstream of the IKK-complex, likely at the level of NOD2.

Next, the effect of siRNA-mediated knock-down of endogenous TRIM27 on NOD2-mediated signaling was determined in THP1 cells that have a functional endogenous NOD2 signaling pathway [46] and express TRIM27 mRNA (Figure S2A). In line with a function of TRIM27 in regulating NOD2 signaling, although not significant, a tendency towards higher MDP-induced IL-8 secretion was observed with two different siRNAs tested (data not shown). This suggests that other described negative regulators of NOD2 can overcome missing regulation by TRIM27. Finally, to explore if TRIM27 expression might be linked to NOD2-associated diseases *in vivo*, we conducted immunohistological inspection of colon sections derived from healthy and active Crohn's disease (CD) patients. This showed that TRIM27 was well expressed in intestinal epithelial cells and to a lesser extent in cells of the lamina propria. In all these cells TRIM27 exhibited a primarily nuclear localization in these cells and appeared to be restricted to sub-nuclear compartments (Figure 6D). Of note, the TRIM27 signal appeared lower in healthy tissue compared to tissue derived from active Crohn's patients (Figure 6D). Analysis of TRIM27 mRNA expression in colon biopsy material of Crohn's disease patients confirmed a significant increase in TRIM27 expression in CD compared to healthy controls (Figure 6E).

Collectively, these data support a role for TRIM27 as a specific negative regulator of NOD2-mediated signaling and suggest a implication of TRIM27 in Crohn's disease.

## Discussion

In this paper we report the identification of the E3 ubiquitin ligase TRIM27 as a new interaction partner for NOD2. Mapping studies indicated that NOD2 binds via its NBD domain to the TRIM27 B30.2/PRY-SPRY domain. The B30.2 domain is composed of the ∼61 aa PRY and the ∼140 aa SPRY domain and is most commonly found at the C-terminus of TRIM proteins [18]. The SPRY domain is evolutionarily more ancient as it is found in animals, plants and fungi, whereas the B30.2 domain is only found in vertebrates with an adaptive immune system [47]. In humans, B30.2/PRY-SPRY domains are only present at the C-terminus in TRIM proteins and in butyrophilin-related transmembrane glycoproteins (BTNs), which are receptors of the immunoglobulin superfamily [48]. Of note, the TRIM27 binding site for NOD2 correlates with the findings for other TRIM proteins: TRIM21 binds its interaction partners IRF3 and IRF8 via the B30.2/PRY-SPRY domain [49]–[51] and TRIM25 binds RIG-I via the C-terminal SPRY domain [52]. TRIM20 (also known as pyrin), regulates caspase-1 activation and IL-1β production by interacting with caspase-1 via its PRY-SPRY and with ASC via its PYD domain, respectively [53]–[55]. Mutations in the human TRIM20 PRY-SPRY domain are associated with inherited Familial Mediterranean Fever (FMF). TRIM20 knock-in mice expressing a TRIM20 with the mutant human PRY-SPRY domain reflect the inflammatory phenotype found in patients by forming spontaneous ASC-dependent, NLRP3-independent inflammasomes [56]. This clearly links the PRY-SPRY domain in TRIM20 to immune functions. Another intriguing example underscoring the involvement of PRY-SPRY domains in NLR regulation is present in zebrafish (*Danio rerio*). One class of NLR proteins in *D. rerio* comprises molecules harboring a PRY-SPRY domain fused to the canonical NLR sequence [57]. Of note, PRY-SPRY domains from *D. rerio* NLRs are closely related to zebrafish TRIMs, indicating domain shuffling during evolution [58]. This suggests that TRIM and NLR proteins have been tied together in evolution to function in innate immune responses.

TRIM proteins are involved in the regulation of PRR- and IFN-signaling pathways, typically by acting as ubiquitin ligases. TRIM-mediated ubiquitination events have been documented to both enhance immune response as shown i.e. for TRIM8, TRIM21, TRIM23, TRIM25 and TRIM56 [49], [51], [52], [59]–[61] or inhibit them as shown for TRIM21 and TRIM30-α [50], [62], [63]. We show here that also TRIM27 is involved in regulating PRR-signaling by negatively regulating NOD2. We observed that NOD2 is ubiquitinated by K48-linked ubiquitin chains and that this ubiquitination is enhanced by TRIM27 overexpression, whereas overexpression of an E3 ligase-deficient mutant of TRIM27 (TRIM27 E3) reduces NOD2 ubiquitination (Figure 4).

K48-linked ubiquitination usually targets proteins to degradation [40], which is partly mediated by TRIM proteins in PRR- and IFN-signaling pathways: Mouse TRIM30-α promotes ubiquitination and degradation – although not by proteasomal but rather by lysosomal degradation – of TAB2 and TAB3 thereby inhibiting TLR-induced NF-κB activation [63], whereas TRIM8 decreases SOCS-1 protein levels leading to an inhibition of IFN-γ-signaling repression by SOCS-1 [59]. Ubiquitinated NOD2 is also targeted to proteasomal degradation, as inhibition of the 26S proteasome with bortezomib led to NOD2 protein stability and accumulation of ubiquitinated NOD2 (Figure 4B). Of note, NOD2 protein stability is effectively dependent on TRIM27. This is evidenced by the finding that knock-down of endogenous TRIM27 as well as overexpression of TRIM27 E3 inhibited NOD2 degradation (Figure 4A and 4C). The latter indicates that TRIM27 E3 acts as dominant negative over WT TRIM27 and endogenous TRIM27. We were surprised that overexpression of TRIM27 did not correlate with reduced NOD2 protein stability. Our interpretation is that this might be due to the activity of endogenous TRIM27 that might be sufficient to target even ectopically expressed NOD2 efficiently for degradation or that degradation at the proteasome is rate-limiting for overexpressed NOD2. TRIM27 has also been shown to confer SUMO E3 ligase activity [29]. However, we did not detect SUMOylated NOD2, suggesting that NOD2 might not be a substrate for TRIM27-mediated SUMOylation (data not shown). In most cases, proteins are ubiquitinated in the cytosol and are degraded in this compartment. However, also evidence for nuclear ubiquitination and protein degradation exists [64]. Based on our results showing that only a small fraction of NOD2 seems to shuttle to the nucleus (Figure 5) we suggest that NOD2 might be ubiquitinated in the cytoplasm.

Regulation of PRRs by ubiquitination and proteasomal degradation is a common theme for PRRs in animals and plants: Triad3A is known to control TLR4 and TLR9 signaling [65] and plant FLS2 has been shown to be ubiquitinated and degraded in a negative feedback-loop by PUB12/13 [66]. Our data suggest that NOD2 signaling is regulated in a similar manner. Indeed, gene reporter assays in HEK293T cells showed that TRIM27 overexpression specifically reduces NOD2-mediated MDP-induced NF-κB activation in a dose-dependent manner, whereas TNF- and IKK-β-induced NF-κB activation is not affected. The negative regulating effects of TRIM27 on NOD2 signaling are not necessarily explained by NOD2 degradation but might also be due to circumvention of adaptor protein recruitment to the NOD2 complex. However, TRIM27 did not inhibit NOD2-RIP2 interaction (Figure S4A). In contrast to our findings, Zha et al. reported that TRIM27 interacts with IKK-α, IKK-β, IKK-ε and negatively regulates NF-κB, IFN-β and ISRE activation induced by these kinases in a RING-domain-independent manner [36]. The discrepancies between the two data sets remain unclear and need further independent investigation.

In contrast to most TRIM proteins, TRIM27 expression is not altered in response to IFNs [16], [17]. Among the TRIM proteins induced by type I and II IFNs, many have been shown to confer antiviral activity, although IFN-inducibility is not a prerequisite for this [17], [39]. Constitutively expressed TRIM proteins have also been shown to trigger antiviral immune responses. TRIM27 overexpression, however, did not influence Sendai virus-induced IFN-β activation (Figure S4B). We therefore assume that TRIM27 might predominantly function in other pathways including NOD2 signaling.

NOD2 has been reported to be localized in the cytosol and at the cell membrane at steady-state conditions. Unexpectedly, we found that ectopically expressed NOD2, in contrast to NOD1, can shuttle to the nucleus (Figure 5), dependently on a functional Walker A motif, i.e. ATPase activity. Although, a strong bipartite nuclear localization signal (NLS) in the NOD2 sequence could not be identified. Due to the low expression of endogenous NOD2, we were not able to establish the sub-cellular localization of endogenous NOD2. So although nuclear shuttling was verified for both NOD2 and epitope-tagged versions of NOD2 ectopically expressed in different cells by both cell biological and biochemical techniques, we cannot formally exclude that this is a particularity of ectopically expressed NOD2. Of note, also two other members of the human NLR family are known to shuttle to the nucleus. CIITA, one of the founding members of the NLR family, has long been recognized to regulate MHC class II gene expression by acting as a scaffold for DNA-binding transcription factor assembly in the nucleus [67], [68]. Recently, also NLRC5 has been shown to localize to the nucleus and to regulate MHC class I expression [44], [45], [69]. Of note, nuclear translocation and functionality are also shown for several NLR-related plant R proteins. For tobacco N, barley MLA, arabidopsis RPS4 and potato RX R proteins nuclear localization is essential for defense activation [70]–[73]. For these it has been suggested that pathogen recognition takes place in the cytoplasm, whereas they act in the nucleus to induce transcriptional reprogramming to activate immune responses [74]. This is exemplified by the tobacco N protein, which confers resistance to Tobacco mosaic virus (TMV). Here MAP kinase activation in the cytosol is necessary for N-mediated resistance in addition to its nuclear functions [75], [76]. In analogy, it is tempting to speculate if NOD2 might not only recognize pathogens and activate MAP kinases and NF-κB in the cytosol, but likely possess additional nuclear functions to mediate and control immune responses. TRIM27 is known to be recruited to specific subnuclear compartments [33] and was suggested as a transcriptional repressor [34], [35]. This raises the intriguing possibility that TRIM27, in addition to its role in NOD2 regulation, might be functionally involved in orchestrating NOD2 nuclear functions. Future research might help to address these open questions.

We and others recently demonstrated that some mutations in the NBD domain of NOD2 can result in enhanced NF-κB activation, whereas the corresponding mutations do not increase the activity of NOD1 [77]. This suggests that NOD2 is more prone for autoactivation compared to NOD1, requesting tighter control mechanisms to act on NOD2 to prevent unwanted auto-inflammatory responses. This is in line with the findings that I) more negative regulators are reported for NOD2 than for NOD1 [78], [79], II) there are more disease-associated mutations found in NOD2 than in NOD1 [11], and III) NOD1 is expressed at basal levels in most tissues whereas NOD2 expression is more restricted and can be induced by pathogen stimuli [8], [80]–[84]. Here, we add another difference between NOD1 and NOD2 regulation and show that NOD2, but not NOD1, is controlled by ubiquitination and subsequent proteasomal degradation dependent on TRIM27 E3 ligase activity. We identified TRIM27 as a negative regulator of NOD2-mediated inflammatory responses and detected enhanced TRIM27 expression in the colon of Crohn's disease patients. Clinically, this makes TRIM27 an interesting new target for NOD2-associated diseases. It is conceivable that targeting TRIM27 might be advantageous when NOD2 activity is altered, such as in Crohn's disease.

## Materials and Methods

### Plasmids

Plasmids encoding N-terminally Flag-tagged NOD1, NOD2 and deletion constructs of NOD2 (ΔCARD: aa250–1040; ΔLRR: aa1–600; CARD: aa1–250; NBD: aa250–600; LRR: aa600–1040) have been described [4], [85]. Myc-NOD1 WT, myc-NOD2 WT and K305R were obtained by subcloning (BamHI-XhoI) into a modified pcDNA3.1 vector containing an N-terminal 3xmyc tag. Human TRIM27 cDNA was obtained from ImaGenes (Berlin, Germany) (GI 115387097). Full length TRIM27 and deletion constructs (ΔRING: aa63–513; ΔRING+BBox: aa133–513; ΔPRY-SPRY: aa1–295; PRY-SPRY: aa296–513) were generated by PCR and cloned into pGEX-II (kindly provided by I. Roux, Institute Pasteur, Paris) or a modified pcDNA3.1 vector (see above). Point mutations of myc-TRIM27 E3 ligase mutant (CC16/31AA) were generated by PCR mutagenesis according to the QuikChange Site-Directed Mutagenesis procedure (Stratagene). IFN-β luciferase reporter was a kind gift from M. Schröder (NUI Maynooth), IgK luciferase NF-κB-reporter and β-galactosidase expression plasmid were described previously [4]. VSV-tagged RIP2 was a kind gift from M. Thome (University Lausanne). The IKK-β plasmid was purchased from InvivoGen. pRK5-HA-ubiquitin (Addgene plasmid 17608) is described in [86]. All constructs were subjected to full-length DNA sequencing.

### Two-hybrid analysis

Binary two-hybrid screening was performed as described by Gyuris et al. [87]. Briefly, the bait plasmids (pEG202) express the cDNA fused directionally to the first 202 residues of LexA under the control of the constitutive ADH promoter. Prey plasmids (pJG4-5) express the cDNA fused to the B42 activation domain, the SV40T nuclear localization signal and an HA-tag under the control of the inducible GAL1 promoter. EGY42/EGY48 diploid strain was generated by mating for every pairwise combination. Six LexA-operators lacZ in the pSH18-34 vector and a genome integrated 4 LexA-operators LEU2 are used as reporters. The expression of two reporters was used to establish the interaction, blue-turning colonies and growth in the absence of Leu.

### Cell culture and siRNA

HEK293T cells were kindly provided by the Sansonetti lab (Institute Pasteur, Paris) [4], HeLa (ATCC #CCL-2) and SW480 (ATCC #CCL-228) cells were obtained from the ATCC. Cells were cultivated at 37°C in a 5% CO_2_ atmosphere in Dulbecco's modified Eagle's medium (Biochrom AG) supplemented with 10% heat-inactivated fetal bovine serum (Biowest), penicillin and streptomycin (100 IU/ml and 100 mg/ml, respectively; Biochrom AG). Cells were continuously tested for absence of mycoplasma contamination by PCR.

All siRNA duplexes were obtained from Qiagen: siCTR (AllStars Negative Control, #1027281); siTRIM27-1 (target sequence AAGACTCAGTGTGCAGAAAAG); siTRIM27-3 (target sequence CAGAACCAGCTCGACCATTTA, Hs_RFP_6, SI03062794); siNOD2 (target sequence CTGCCACATGCAAGAAGTATA; Hs_CARD15_3, SI00133049); siRIP2 (target sequence ACGTATGATCTCTCTAATAGA).

### Co-immunoprecipitation and immunoblotting

For co-immunoprecipitations, HEK293T cells were seeded in 6 cm dishes and were transiently transfected with 1 µg plasmid as indicated using Lipofectamin2000 (Invitrogen) or FuGene6 (Roche). Cells were lysed in NP40 buffer (150 mM NaCl, 50 mM Tris pH 7.4, 1% NP40) or RIPA buffer (150 mM NaCl, 50 mM Tris pH 7.4, 1% Triton X-100, 0.1% SDS, 0.5% deoxycholate) containing phosphatase inhibitors (20 µM β-glycerophosphate, 5 mM NaF, 100 µM Na_3_VO_4_) and protease inhibitors (Complete protease inhibitor cocktail with EDTA; Roche). Lysates were cleared for 20 min at 14,000× g at 4°C. Flag-tagged NOD2 was immunoprecipitated with anti-Flag beads (M2 agarose; Sigma), myc-tagged TRIM27 with anti-myc beads (c-myc AC, clone 9E10; Santa Cruz Biotechnology) for 3 h at 4°C. Beads were washed five times with lysis buffer before SDS loading buffer containing β-mercaptoethanol was added. Typically, 10-times more precipitate than input was loaded onto the gel. Proteins were separated by Laemmli sodium dodecylsulfate-polyacrylamide gel electrophoresis (SDS-PAGE) and subsequently transferred on nitrocellulose membrane (Bio-Rad) by semi-dry immunoblotting. Proteins were detected by incubation of the membrane successive with primary and secondary antibody and a final incubation with SuperSignal West Pico Chemiluminescent Substrate or Femto Maximum Sensitivity Substrate (Pierce). Signals were recorded on an electronic imaging system (LAS4000, Fujifilm). Primary antibodies were mouse anti-Flag M2 (1∶2000; Stratagene), rabbit anti-myc (1∶500; A-14, Santa Cruz Biotechnology), rabbit anti-TRIM27 (1∶400; IBL), rabbit anti-HA (1∶500; Y-11, Santa Cruz Biotechnology), mouse anti-ubiquitin (1∶500; Santa Cruz Biotechnology), mouse anti-ubiquitin K48 (1∶1000; Millipore), mouse anti-ubiquitin K63 (1∶1000; Millipore), rat anti-NOD2 4A11 (1∶100; [4]), rabbit anti-lamin A/C (1∶500; Cell Signaling Technology), rabbit anti-GAPDH (1∶1000; Santa Cruz Biotechnology) and rabbit anti-actin (1∶1000; A2066, Sigma-Aldrich). Secondary antibodies were horseradish peroxidase (HRP)-conjugated goat anti-mouse IgG, light chain specific (1∶4000; Jackson ImmunoResearch Laboratories), HRP-conjugated goat anti-mouse IgG (1∶4000; 170-6516, Bio-Rad), HRP-conjugated goat anti-rabbit IgG (1∶4000; 170-6515, Bio-Rad) and HRP-conjugated goat anti-rat IgG (1∶4000; Jackson ImmunoResearch Laboratories). All antibodies were diluted in 5% milk in PBS or TBS, according to the manufacturer's instructions.

Endogenous NOD2 was immunoprecipitated from SW480 cells lysed in NP40 buffer using the NOD2 specific monoclonal rat antibody 6F6 [6] bound to Protein-G Sepharose beads.

For testing for ubiquitination of NOD2, HEK293T cells were lysed in NP40 buffer containing 1% SDS and boiled for 10 min. Immunoprecipitations were conducted from these samples diluted 1∶10 in NP40 buffer as described above.

### Expression of recombinant GST-TRIM27

TRIM27 and its deletion constructs were expressed as N-terminal glutathione S-transferase (GST) fusion proteins in Rosetta2 (DE3) pLys *E.coli* cells (kindly provided by G. Praefcke) following overnight isopropyl 1-thio-β-d-galactopyranoside (IPTG, 100 µM; Sigma) induction at 20°C. Bacteria were lysed by a freeze-and-thaw cycle followed by addition of 1.5% sarkosyl (Sigma) and sonication. All GST fusion proteins were purified from bacterial extracts with glutathione-Sepharose (Amersham Biosciences), followed by extensive washing with buffer A (150 mM NaCl, 50 mM Tris pH 7.5, 2 mM DTT), buffer B (500 mM NaCl, 50 mM Tris pH 7.5, 2 mM DTT) and again buffer A. TRIM27 protein bound to glutathione-Sepharose was stored in buffer A at −80°C.

### In vitro translation and GST-binding assay

*In vitro* transcription/translation (IVT) of NOD2 was performed from the Flag-NOD2 construct by T7 RNA polymerase (Promega) in the presence of L-[35S]-methionine (10 mCi/ml; Perkin Elmer) using the TnT coupled reticulocyte lysate system (Promega) according to the manufacturer's instructions. IVT preparation was diluted three times in PBS and incubated with recombinant TRIM27 protein bound to glutathione-Sepharose beads for 2 h at 4°C. Beads were precipitated and washed five times with RIPA buffer (150 mM NaCl, 50 mM Tris pH 7.4, 1% Triton, 0.1% SDS, 0.5% deoxycholate) before SDS loading buffer containing β-mercaptoethanol was added. Proteins were analyzed by Laemmli sodium dodecylsulfate-polyacrylamide gel electrophoresis (SDS-PAGE) followed by Coomassie brilliant blue staining. Autoradiographs were recorded using a PhosphorImager (Bio-Rad).

### Indirect immunofluorescence microscopy

For indirect immunofluorescence microscopy, HeLa cells were seeded in 24-well plates on glass coverslips and transiently transfected with 0.8 µg of expression plasmids, as indicated using Lipofectamin2000 transfection reagent (Invitrogen) according to the manufacturer's instructions. After 24 h, cells were fixed with 3% paraformaldehyde (Roth) in PBS for 10 min and permeabilized with 0.5% Triton X-100 (Roth) in cold PBS for 5 min. Cells were blocked in 3% bovine serum albumin (BSA; Roth) in PBS for 20 min and incubated successively in primary and secondary antibodies. Primary antibodies: rabbit anti-TRIM27 (1∶500; IBL) and mouse anti-myc (1∶1000; 9E10, Sigma). Secondary antibody: goat anti-rabbit AlexaFluor 546 (1∶250; Invitrogen Molecular Probes) and goat anti-mouse AlexaFluor 488 (1∶250; Invitrogen Molecular Probes). DNA was stained with DAPI (5 µg/ml; Invitrogen Molecular Probes). Cells were mounted in ProLong Gold antifade reagent (Invitrogen Molecular Probes). Image acquisition of z-stacks was performed on an Olympus FV-1000 laser scanning microscope (objective: Olympus PlanApo, 60×/1.40 oil, ∞/0.17) and images were processed using the ImageJ software [88].

### Sub-cellular fractionation

For fractionation, HEK293T cells were seeded in 10 cm dishes and transiently transfected with 3 µg plasmid as indicated. After 24 h, cellular fractions were prepared using the Qproteome cell compartment kit (Qiagen). Flag-tagged proteins in the different fractions were precipitated using anti-Flag beads as described above.

### Luciferase reporter assay

3×10^4^ HEK293T cells per well were seeded in 96-well plates and transfected with 8.6 ng β-galactosidase, 13 ng luciferase NF-κB- or 10 ng IFN-β-reporter, different amounts of myc-TRIM27 and either with or without 0.1 ng NOD2 or 10 ng IKK-β expression plasmids as indicated, added up with pcDNA to 51 ng total DNA using FuGene6 (Roche). Cells were directly stimulated with 50 nM MDP, 10 ng/ml TNF (all obtained from InvivoGen) or 133 hemagglutination units (HAU)/ml Sendai virus (hen egg allantoid fluid; obtained from Charles River Laboratories) or left unstimulated, as indicated. After 16 h, cells were lysed in luciferase lysis buffer (25 mM Tris pH 8, 8 mM MgCl_2_, 1% Triton, 15% glycerol, 1 mM DTT) and luciferase activity was measured using a standard plate luminometer. Standard deviation (SD) was calculated from triplets and luciferase activity was normalized as a ratio to β-galactosidase activity. All experiments were repeated independently at least three times.

### RT-PCR

End point RT-PCR was performed using *Taq* polymerase (Fermentas) on cDNA obtained from isolated RNA of the indicated cell lines. RNA was isolated using the RNeasy kit (Qiagen) and 1 µg of total RNA was transcribed into cDNA using the First Strand cDNA synthesis kit (Fermentas) with an oligo (dT) primer (Fermentas). Primers for TRIM27 were obtained from Qiagen (QT00051954). For GAPDH, the following primer pair was used: GAPDH_fwd, GGTATCGTGGAAGGACTCATGAC; and GAPDH_rev, ATGCCAGTGAGCTTCCCGTTCAG. The PCR products were separated by agarose gel electrophoresis and visualized using ethidium bromide.

### qRT-PCR of TRIM27 from patient samples

Endoscopic biopsies were taken from the sigmoid colon of patients with mildly to moderately active Crohn's disease and controls. Only patients with no endoscopic or histological evidence of mucosal inflammation were included in the control group. Written informed consent was obtained from all patients before endoscopy. The study was approved by the local ethical committee of the University Hospital Aachen, Germany. Biopsies were immediately shock-frozen in liquid nitrogen and stored at −80°C. RNA from frozen tissue biopsies was isolated using PegGOLD RNAPure (PEQLAB Biotechnology) according to the manufacturer's instructions. 2 µg of RNA were treated with DNAseI (Invitrogen) before cDNA was generated by reverse transcription with 200 ng random hexamer primer (Invitrogen), 500 µM dNTPs (Invitrogen) and 4 U Omniscript Reverse Transcriptase (Quiagen) in 1× reverse transcriptase buffer for 1 h at 37°C. Real-time PCR was performed using Real Time PCR System 7300 (Applied Biosystems) and SYBRGreenER qPCR Super Mix (Invitrogen). TRIM27 expression levels were normalized to GAPDH. Primers for TRIM27 were obtained from Qiagen (QT00051954). For GAPDH, the following primer pair was used: GAPDH_fwd, CAGCCTCAAGATCATCAGCA; and GAPDH_rev, CCTTCCACGATACCAAAGTTGTC.

### Immunfluorescence microscopy of human specimens

Colon biopsies from Crohn's disease and control patients were obtained during colonoscopy surgeries and placed in Tissue-Tek® (Sakura Finetek Europe B.V, Netherlands). Frozen samples were cut in 7 µm-sections and fixed with 4% paraformaldehyde for 15 minutes at 4°C. Slides were blocked for 1 hour with 50% (v/v) fetal calf serum and 50% (v/v) PBS/BSA 1% at room temperature. To assess TRIM27 expression anti-RET Finger Protein (RFP) rabbit IgG affinity purified primary antibody (0.1 µg/ml, IBL, Japan) and secondary Alexa Fluor 594 goat anti-rabbit IgG (H+L) (1∶200 dilution, Invitrogen, USA) were used. F-Actin-staining was performed by using Phalloidin FITC labeled mixed isomers (2 µg/ml in DMSO, Sigma-Aldrich). Slides were mounted with Vectashield/DAPI stain (Vector Laboratories H-1200) and stored at 4°C in darkness prior to immunfluorescence microscopy. Images were captured with Zeiss AxioVision LE Rel. 4.4 on a Zeiss Axio Imager Z1 (Carl Zeiss Inc.) with a 20× objective.

### Statistical analysis

Data are presented as mean+SD. Significance was assessed with the two-sample Student's t-test (*, P<0.05; **, P<0.01; ***, P<0.005)

## Supporting Information

Figure S1 **Screening for TRIM proteins interacting with NOD2.** *A.* Results obtained from the Y2H screen using human NOD2 full length (NOD2 fl) and several human TRIM proteins as bait and prey, as indicated. x, weak interaction; “XX”, strong interaction. *B.* Characterization of the rat 6F6 anti-NOD2 antibody. Western blot probed with 6F6, loaded with different amounts of whole cell lysates from SW480 cells and SW480 cells treated with a NOD2 specific siRNA for 48 h is shown.
(TIF)

Figure S2 **TRIM27 mRNA expression and cellular localization.** *A.* End-point RT-PCR analysis of TRIM27 mRNA expression in different cell lines. Amplification of GAPDH served as control. *B.* Indirect immunofluorescence micrographs of HeLa and HEK cells grown on coverslips. Images with signals for DAPI, TRIM27 and an overlay (blue: DAPI, red: TRIM27) are shown. *C.* HeLa cells were stimulated with 10 µM MDP for 3 h or left unstimulated. Cellular fractions were prepared using the Qproteome cell compartment kit. Immunoblot analysis was performed using the indicated antibodies. *D.* Indirect immunofluorescence micrographs of HeLa cells grown on coverslips and expressing myc-NOD1 were treated with 50 nM LMB for 4 h or left untreated. Images with signals for DAPI, myc-NOD1 and an overlay (blue: DAPI, green: NOD1) are shown. Bars, 10 µm.
(TIF)

Figure S3 **NOD2 but not NOD1 is degraded.** *A.* HEK cells expressing Flag-NOD1 or –NOD2 were treated with 30 µg/ml CHX, as indicated. Immunoblots of total cell lysates (top) were performed using the indicated antibodies. GAPDH served as loading control. Densitometric analysis (bottom) of the NOD1 and NOD2 signals normalized to GAPDH is shown. *B.* HEK293T cells transfected with Flag-NOD2 and myc-TRIM27, E3 or CTR as indicated were treated with 30 µg/ml cycloheximid (CHX) and immunoblots of total cell lysates (top) were performed using the indicated antibodies. GAPDH served as loading control (related to Figure 4A).
(TIF)

Figure S4 **Effect of TRIM27 on RIP2/Nod2 interaction and IFN signalling.** *A.* Lysates of HEK cells expressing the indicated proteins were subjected to immunoprecipitation using anti-Flag beads. Immunoblots of immunoprecipitates (IP) and total lysates (Input) were performed using the indicated antibodies. *B.* To determine the influence of TRIM27 on Sendai virus-induced IFN-β promoter activation, HEK293T cells were transfected with different amounts of TRIM27, as indicated, and an IFN-β promotor luciferase reporter system. Cells were then infected with 133 HAU/ml Sendai virus. Normalized luciferase activity (nRLU) is shown. Values are given as mean+SD.
(TIF)

## References

[1] Ting JP, Duncan JA, Lei Y (2010). How the noninflammasome NLRs function in the innate immune system.. Science.

[2] Chen G, Shaw MH, Kim YG, Nunez G (2009). NOD-like receptors: role in innate immunity and inflammatory disease.. Annu Rev Pathol.

[3] Magalhaes JG, Sorbara MT, Girardin SE, Philpott DJ (2011). What is new with Nods?. Curr Opin Immunol.

[4] Kufer TA, Kremmer E, Banks DJ, Philpott DJ (2006). Role for erbin in bacterial activation of Nod2.. Infect Immun.

[5] Barnich N, Aguirre JE, Reinecker HC, Xavier R, Podolsky DK (2005). Membrane recruitment of NOD2 in intestinal epithelial cells is essential for nuclear factor-{kappa}B activation in muramyl dipeptide recognition.. J Cell Biol.

[6] Legrand-Poels S, Kustermans G, Bex F, Kremmer E, Kufer TA (2007). Modulation of Nod2-dependent NF-kappaB signaling by the actin cytoskeleton.. J Cell Sci.

[7] Philpott DJ, Girardin SE (2010). Nod-like receptors: sentinels at host membranes.. Curr Opin Immunol.

[8] Ogura Y, Inohara N, Benito A, Chen FF, Yamaoka S (2001). Nod2, a Nod1/Apaf-1 family member that is restricted to monocytes and activates NF-kappaB.. J Biol Chem.

[9] Kobayashi K, Inohara N, Hernandez LD, Galan JE, Nunez G (2002). RICK/Rip2/CARDIAK mediates signalling for receptors of the innate and adaptive immune systems.. Nature.

[10] Kobayashi KS, Chamaillard M, Ogura Y, Henegariu O, Inohara N (2005). Nod2-dependent regulation of innate and adaptive immunity in the intestinal tract.. Science.

[11] Borzutzky A, Fried A, Chou J, Bonilla FA, Kim S (2009). NOD2-associated diseases: Bridging innate immunity and autoinflammation.. Clin Immunol.

[12] McDonald C, Chen FF, Ollendorff V, Ogura Y, Marchetto S (2005). A role for Erbin in the regulation of Nod2-dependent NF-kappaB signaling.. J Biol Chem.

[13] Yamamoto-Furusho JK, Barnich N, Xavier R, Hisamatsu T, Podolsky DK (2006). Centaurin beta1 down-regulates nucleotide-binding oligomerization domains 1- and 2-dependent NF-kappaB activation.. J Biol Chem.

[14] Bielig H, Zurek B, Kutsch A, Menning M, Philpott DJ (2009). A function for AAMP in Nod2-mediated NF-kappaB activation.. Mol Immunol.

[15] Sardiello M, Cairo S, Fontanella B, Ballabio A, Meroni G (2008). Genomic analysis of the TRIM family reveals two groups of genes with distinct evolutionary properties.. BMC Evol Biol.

[16] Rajsbaum R, Stoye JP, O'Garra A (2008). Type I interferon-dependent and -independent expression of tripartite motif proteins in immune cells.. Eur J Immunol.

[17] Carthagena L, Bergamaschi A, Luna JM, David A, Uchil PD (2009). Human TRIM gene expression in response to interferons.. PLoS One.

[18] Ozato K, Shin DM, Chang TH, Morse HC (2008). TRIM family proteins and their emerging roles in innate immunity.. Nat Rev Immunol.

[19] McNab FW, Rajsbaum R, Stoye JP, O'Garra A (2011). Tripartite-motif proteins and innate immune regulation.. Curr Opin Immunol.

[20] Bailly V, Lauder S, Prakash S, Prakash L (1997). Yeast DNA repair proteins Rad6 and Rad18 form a heterodimer that has ubiquitin conjugating, DNA binding, and ATP hydrolytic activities.. J Biol Chem.

[21] Deshaies RJ, Joazeiro CA (2009). RING domain E3 ubiquitin ligases.. Annu Rev Biochem.

[22] French FMF Consortium (1997). A candidate gene for familial Mediterranean fever.. Nat Genet.

[23] International FMF Consortium (1997). Ancient missense mutations in a new member of the RoRet gene family are likely to cause familial Mediterranean fever. The International FMF Consortium.. Cell.

[24] Harley JB, Alexander EL, Bias WB, Fox OF, Provost TT (1986). Anti-Ro (SS-A) and anti-La (SS-B) in patients with Sjogren's syndrome.. Arthritis Rheum.

[25] Ishii T, Ohnuma K, Murakami A, Takasawa N, Yamochi T (2003). SS-A/Ro52, an autoantigen involved in CD28-mediated IL-2 production.. J Immunol.

[26] Nakayama EE, Shioda T (2010). Anti-retroviral activity of TRIM5 alpha.. Rev Med Virol.

[27] Hasegawa N, Iwashita T, Asai N, Murakami H, Iwata Y (1996). A RING finger motif regulates transforming activity of the rfp/ret fusion gene.. Biochem Biophys Res Commun.

[28] Napolitano LM, Jaffray EG, Hay RT, Meroni G (2011). Functional interactions between ubiquitin E2 enzymes and TRIM proteins.. Biochem J.

[29] Chu Y, Yang X (2011). SUMO E3 ligase activity of TRIM proteins.. Oncogene.

[30] Tezel G, Nagasaka T, Iwahashi N, Asai N, Iwashita T (1999). Different nuclear/cytoplasmic distributions of RET finger protein in different cell types.. Pathol Int.

[31] Cao T, Borden KL, Freemont PS, Etkin LD (1997). Involvement of the rfp tripartite motif in protein-protein interactions and subcellular distribution.. J Cell Sci.

[32] Cao T, Duprez E, Borden KL, Freemont PS, Etkin LD (1998). Ret finger protein is a normal component of PML nuclear bodies and interacts directly with PML.. J Cell Sci.

[33] Matsuura T, Shimono Y, Kawai K, Murakami H, Urano T (2005). PIAS proteins are involved in the SUMO-1 modification, intracellular translocation and transcriptional repressive activity of RET finger protein.. Exp Cell Res.

[34] Shimono Y, Murakami H, Hasegawa Y, Takahashi M (2000). RET finger protein is a transcriptional repressor and interacts with enhancer of polycomb that has dual transcriptional functions.. J Biol Chem.

[35] Bloor AJ, Kotsopoulou E, Hayward P, Champion BR, Green AR (2005). RFP represses transcriptional activation by bHLH transcription factors.. Oncogene.

[36] Zha J, Han KJ, Xu LG, He W, Zhou Q (2006). The Ret finger protein inhibits signaling mediated by the noncanonical and canonical IkappaB kinase family members.. J Immunol.

[37] Dho SH, Kwon KS (2003). The Ret finger protein induces apoptosis via its RING finger-B box-coiled-coil motif.. J Biol Chem.

[38] Patel CA, Ghiselli G (2005). The RET finger protein interacts with the hinge region of SMC3.. Biochem Biophys Res Commun.

[39] Reymond A, Meroni G, Fantozzi A, Merla G, Cairo S (2001). The tripartite motif family identifies cell compartments.. Embo J.

[40] Hershko A, Ciechanover A (1998). The ubiquitin system.. Annu Rev Biochem.

[41] Benko S, Magalhaes JG, Philpott DJ, Girardin SE (2010). NLRC5 limits the activation of inflammatory pathways.. J Immunol.

[42] Cressman DE, Chin KC, Taxman DJ, Ting JP (1999). A defect in the nuclear translocation of CIITA causes a form of type II bare lymphocyte syndrome.. Immunity.

[43] Harton JA, Cressman DE, Chin KC, Der CJ, Ting JP (1999). GTP binding by class II transactivator: role in nuclear import.. Science.

[44] Meissner TB, Li A, Biswas A, Lee KH, Liu YJ (2010). NLR family member NLRC5 is a transcriptional regulator of MHC class I genes.. Proc Natl Acad Sci U S A.

[45] Neerincx A, Rodriguez GM, Steimle V, Kufer TA (2012). NLRC5 Controls Basal MHC Class I Gene Expression in an MHC Enhanceosome-Dependent Manner.. J Immunol.

[46] Uehara A, Yang S, Fujimoto Y, Fukase K, Kusumoto S (2005). Muramyldipeptide and diaminopimelic acid-containing desmuramylpeptides in combination with chemically synthesized Toll-like receptor agonists synergistically induced production of interleukin-8 in a NOD2- and NOD1-dependent manner, respectively, in human monocytic cells in culture.. Cell Microbiol.

[47] Rhodes DA, de Bono B, Trowsdale J (2005). Relationship between SPRY and B30.2 protein domains. Evolution of a component of immune defence?. Immunology.

[48] Henry J, Ribouchon M, Depetris D, Mattei M, Offer C (1997). Cloning, structural analysis, and mapping of the B30 and B7 multigenic families to the major histocompatibility complex (MHC) and other chromosomal regions.. Immunogenetics.

[49] Kong HJ, Anderson DE, Lee CH, Jang MK, Tamura T (2007). Cutting edge: autoantigen Ro52 is an interferon inducible E3 ligase that ubiquitinates IRF-8 and enhances cytokine expression in macrophages.. J Immunol.

[50] Higgs R, Ni Gabhann J, Ben Larbi N, Breen EP, Fitzgerald KA (2008). The E3 ubiquitin ligase Ro52 negatively regulates IFN-beta production post-pathogen recognition by polyubiquitin-mediated degradation of IRF3.. J Immunol.

[51] Yang K, Shi HX, Liu XY, Shan YF, Wei B (2009). TRIM21 is essential to sustain IFN regulatory factor 3 activation during antiviral response.. J Immunol.

[52] Gack MU, Shin YC, Joo CH, Urano T, Liang C (2007). TRIM25 RING-finger E3 ubiquitin ligase is essential for RIG-I-mediated antiviral activity.. Nature.

[53] Richards N, Schaner P, Diaz A, Stuckey J, Shelden E (2001). Interaction between pyrin and the apoptotic speck protein (ASC) modulates ASC-induced apoptosis.. J Biol Chem.

[54] Chae JJ, Wood G, Masters SL, Richard K, Park G (2006). The B30.2 domain of pyrin, the familial Mediterranean fever protein, interacts directly with caspase-1 to modulate IL-1beta production.. Proc Natl Acad Sci U S A.

[55] Chae JJ, Komarow HD, Cheng J, Wood G, Raben N (2003). Targeted disruption of pyrin, the FMF protein, causes heightened sensitivity to endotoxin and a defect in macrophage apoptosis.. Mol Cell.

[56] Chae JJ, Cho YH, Lee GS, Cheng J, Liu PP (2011). Gain-of-Function Pyrin Mutations Induce NLRP3 Protein-Independent Interleukin-1beta Activation and Severe Autoinflammation in Mice.. Immunity.

[57] Laing KJ, Purcell MK, Winton JR, Hansen JD (2008). A genomic view of the NOD-like receptor family in teleost fish: identification of a novel NLR subfamily in zebrafish.. BMC Evol Biol.

[58] van der Aa LM, Levraud JP, Yahmi M, Lauret E, Briolat V (2009). A large new subset of TRIM genes highly diversified by duplication and positive selection in teleost fish.. BMC Biol.

[59] Toniato E, Chen XP, Losman J, Flati V, Donahue L (2002). TRIM8/GERP RING finger protein interacts with SOCS-1.. J Biol Chem.

[60] Arimoto K, Funami K, Saeki Y, Tanaka K, Okawa K (2010). Polyubiquitin conjugation to NEMO by triparite motif protein 23 (TRIM23) is critical in antiviral defense.. Proc Natl Acad Sci U S A.

[61] Tsuchida T, Zou J, Saitoh T, Kumar H, Abe T (2010). The ubiquitin ligase TRIM56 regulates innate immune responses to intracellular double-stranded DNA.. Immunity.

[62] Hu Y, Mao K, Zeng Y, Chen S, Tao Z (2010). Tripartite-motif protein 30 negatively regulates NLRP3 inflammasome activation by modulating reactive oxygen species production.. J Immunol.

[63] Shi M, Deng W, Bi E, Mao K, Ji Y (2008). TRIM30 alpha negatively regulates TLR-mediated NF-kappa B activation by targeting TAB2 and TAB3 for degradation.. Nat Immunol.

[64] von Mikecz A (2006). The nuclear ubiquitin-proteasome system.. J Cell Sci.

[65] Chuang TH, Ulevitch RJ (2004). Triad3A, an E3 ubiquitin-protein ligase regulating Toll-like receptors.. Nat Immunol.

[66] Lu D, Lin W, Gao X, Wu S, Cheng C (2011). Direct ubiquitination of pattern recognition receptor FLS2 attenuates plant innate immunity.. Science.

[67] Steimle V, Otten LA, Zufferey M, Mach B (1993). Complementation cloning of an MHC class II transactivator mutated in hereditary MHC class II deficiency (or bare lymphocyte syndrome).. Cell.

[68] Zika E, Ting JP (2005). Epigenetic control of MHC-II: interplay between CIITA and histone-modifying enzymes.. Curr Opin Immunol.

[69] Staehli F, Ludigs K, Heinz LX, Seguin-Estevez Q, Ferrero I (2012). NLRC5 deficiency selectively impairs MHC class I- dependent lymphocyte killing by cytotoxic T cells.. J Immunol.

[70] Tameling WI, Baulcombe DC (2007). Physical association of the NB-LRR resistance protein Rx with a Ran GTPase-activating protein is required for extreme resistance to Potato virus X.. Plant Cell.

[71] Wirthmueller L, Zhang Y, Jones JD, Parker JE (2007). Nuclear accumulation of the Arabidopsis immune receptor RPS4 is necessary for triggering EDS1-dependent defense.. Curr Biol.

[72] Burch-Smith TM, Schiff M, Caplan JL, Tsao J, Czymmek K (2007). A novel role for the TIR domain in association with pathogen-derived elicitors.. PLoS Biol.

[73] Shen QH, Saijo Y, Mauch S, Biskup C, Bieri S (2007). Nuclear activity of MLA immune receptors links isolate-specific and basal disease-resistance responses.. Science.

[74] Liu J, Coaker G (2008). Nuclear trafficking during plant innate immunity.. Mol Plant.

[75] Liu Y, Jin H, Yang KY, Kim CY, Baker B (2003). Interaction between two mitogen-activated protein kinases during tobacco defense signaling.. Plant J.

[76] Jin H, Liu Y, Yang KY, Kim CY, Baker B (2003). Function of a mitogen-activated protein kinase pathway in N gene-mediated resistance in tobacco.. Plant J.

[77] Zurek B, Proell M, Wagner RN, Schwarzenbacher R, Kufer TA (2012). Mutational analysis of human NOD1 and NOD2 NACHT domains reveals different modes of activation.. Innate Immun.

[78] Lecat A, Piette J, Legrand-Poels S (2010). The protein Nod2: an innate receptor more complex than previously assumed.. Biochem Pharmacol.

[79] Kufer TA (2008). Signal transduction pathways used by NLR-type innate immune receptors.. Mol Biosyst.

[80] Sabbah A, Chang TH, Harnack R, Frohlich V, Tominaga K (2009). Activation of innate immune antiviral responses by Nod2.. Nat Immunol.

[81] Oh HM, Lee HJ, Seo GS, Choi EY, Kweon SH (2005). Induction and localization of NOD2 protein in human endothelial cells.. Cell Immunol.

[82] Rosenstiel P, Fantini M, Brautigam K, Kuhbacher T, Waetzig GH (2003). TNF-alpha and IFN-gamma regulate the expression of the NOD2 (CARD15) gene in human intestinal epithelial cells.. Gastroenterology.

[83] Gutierrez O, Pipaon C, Inohara N, Fontalba A, Ogura Y (2002). Induction of Nod2 in myelomonocytic and intestinal epithelial cells via nuclear factor-kappa B activation.. J Biol Chem.

[84] Ogura Y, Lala S, Xin W, Smith E, Dowds TA (2003). Expression of NOD2 in Paneth cells: a possible link to Crohn's ileitis.. Gut.

[85] Kufer TA, Kremmer E, Adam AC, Philpott DJ, Sansonetti PJ (2008). The pattern-recognition molecule Nod1 is localized at the plasma membrane at sites of bacterial interaction.. Cell Microbiol.

[86] Lim KL, Chew KC, Tan JM, Wang C, Chung KK (2005). Parkin mediates nonclassical, proteasomal-independent ubiquitination of synphilin-1: implications for Lewy body formation.. J Neurosci.

[87] Gyuris J, Golemis E, Chertkov H, Brent R (1993). Cdi1, a human G1 and S phase protein phosphatase that associates with Cdk2.. Cell.

[88] Rasband WS (2007). ImageJ.

