# Supplementary figures and images for: TRIM27 Negatively Regulates NOD2 by Ubiquitination and Proteasomal Degradation
Source: PLoS One. 2012 Jul 19;7(7):e41255. doi: 10.1371/journal.pone.0041255 (PMC3400628; doi:10.1371/journal.pone.0041255)

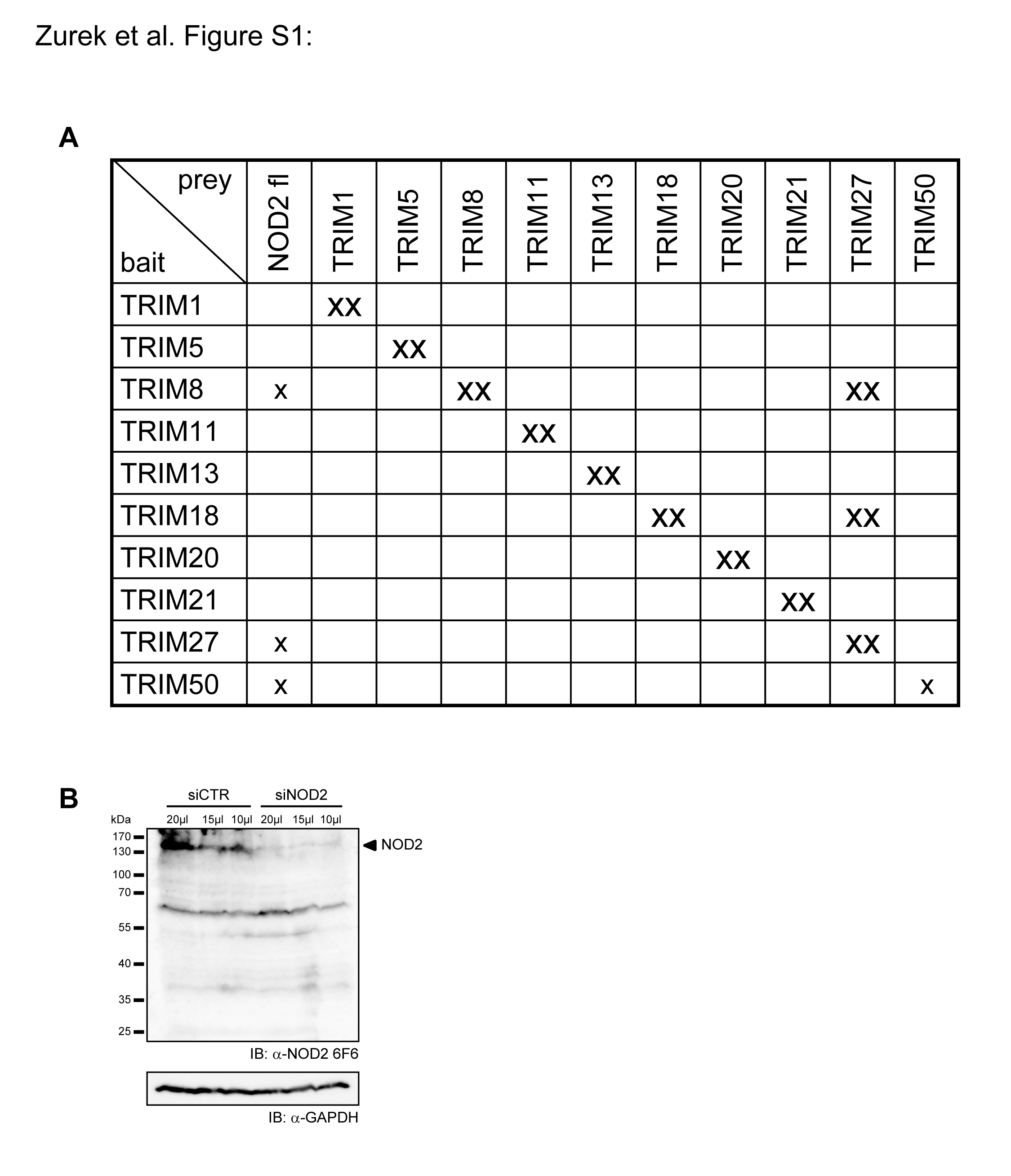

Supplement: Figure S1 — Screening for TRIM proteins interacting with NOD2. A. Results obtained from the Y2H screen using human NOD2 full length (NOD2 fl) and several human TRIM proteins as bait and prey, as indicated. x, weak interaction; “XX”, strong interaction. B. Characterization of the rat 6F6 anti-NOD2 antibody. Western blot probed with 6F6, loaded with different amounts of whole cell lysates from SW480 cells and SW480 cells treated with a NOD2 specific siRNA for 48 h is shown. (TIF) [file pone.0041255.s001.tif]

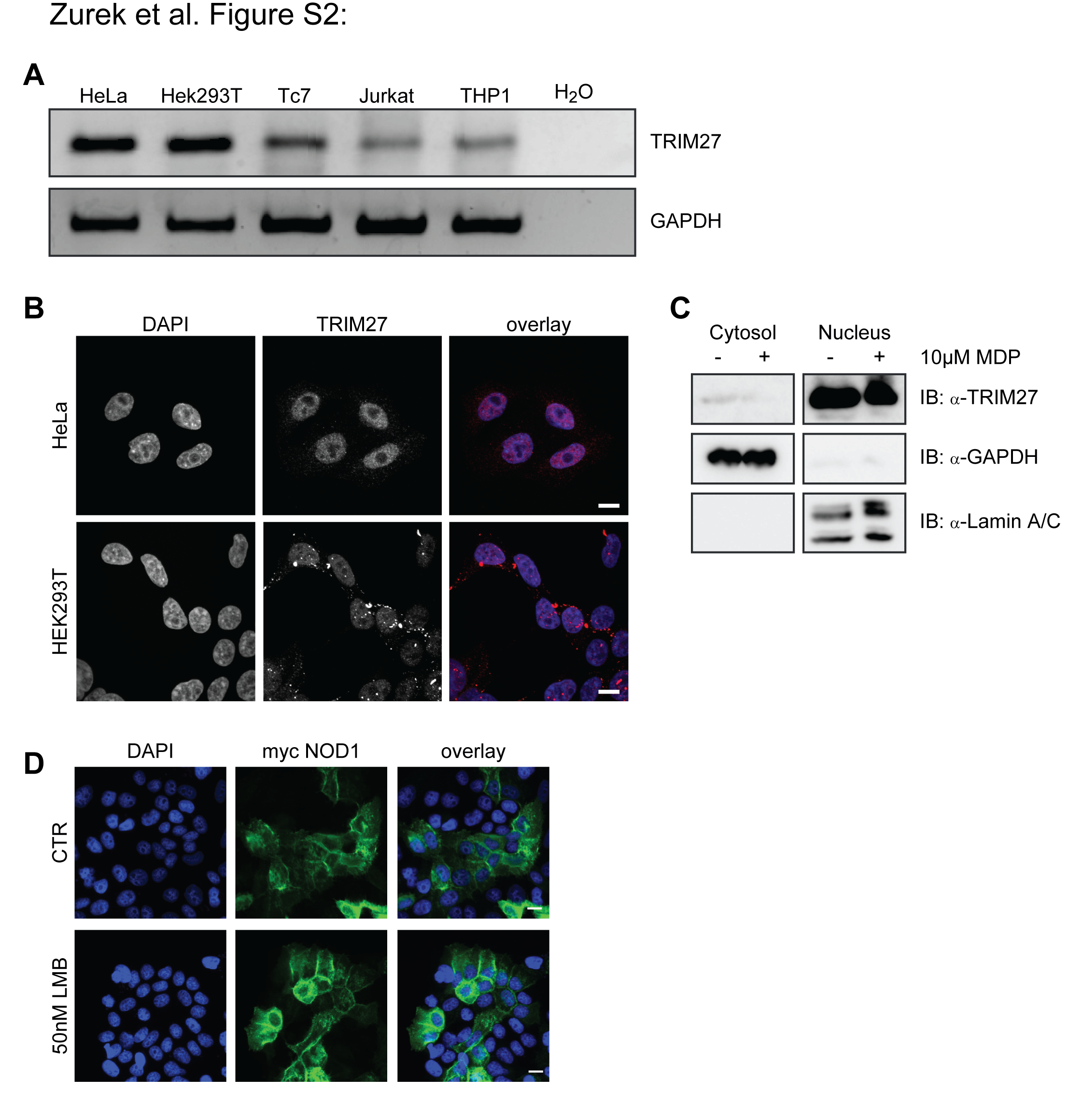

Supplement: Figure S2 — TRIM27 mRNA expression and cellular localization. A. End-point RT-PCR analysis of TRIM27 mRNA expression in different cell lines. Amplification of GAPDH served as control. B. Indirect immunofluorescence micrographs of HeLa and HEK cells grown on coverslips. Images with signals for DAPI, TRIM27 and an overlay (blue: DAPI, red: TRIM27) are shown. C. HeLa cells were stimulated with 10 µM MDP for 3 h or left unstimulated. Cellular fractions were prepared using the Qproteome cell compartment kit. Immunoblot analysis was performed using the indicated antibodies. D. Indirect immunofluorescence micrographs of HeLa cells grown on coverslips and expressing myc-NOD1 were treated with 50 nM LMB for 4 h or left untreated. Images with signals for DAPI, myc-NOD1 and an overlay (blue: DAPI, green: NOD1) are shown. Bars, 10 µm. (TIF) [file pone.0041255.s002.tif]

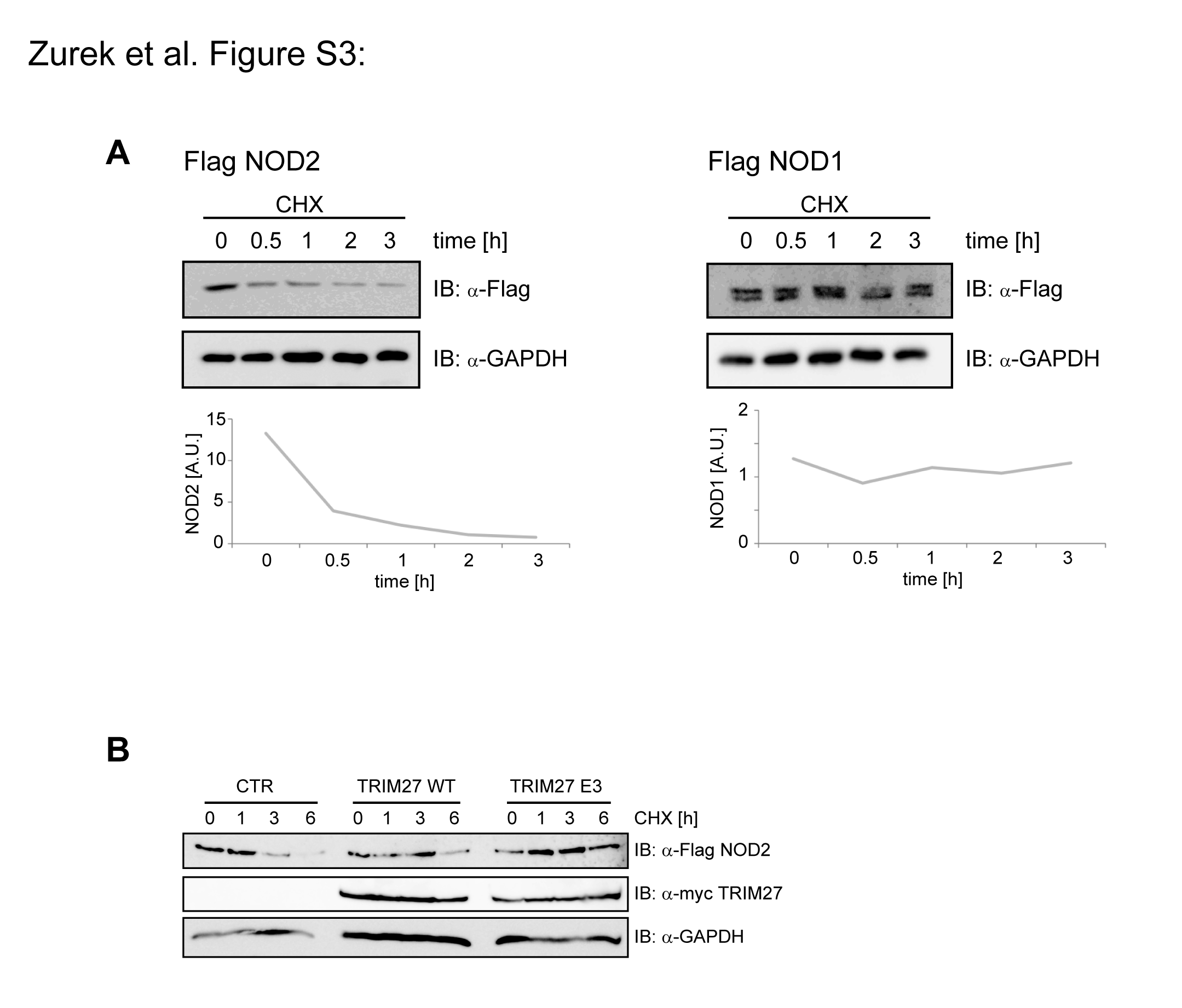

Supplement: Figure S3 — NOD2 but not NOD1 is degraded. A. HEK cells expressing Flag-NOD1 or –NOD2 were treated with 30 µg/ml CHX, as indicated. Immunoblots of total cell lysates (top) were performed using the indicated antibodies. GAPDH served as loading control. Densitometric analysis (bottom) of the NOD1 and NOD2 signals normalized to GAPDH is shown. B. HEK293T cells transfected with Flag-NOD2 and myc-TRIM27, E3 or CTR as indicated were treated with 30 µg/ml cycloheximid (CHX) and immunoblots of total cell lysates (top) were performed using the indicated antibodies. GAPDH served as loading control (related to Figure 4A). (TIF) [file pone.0041255.s003.tif]

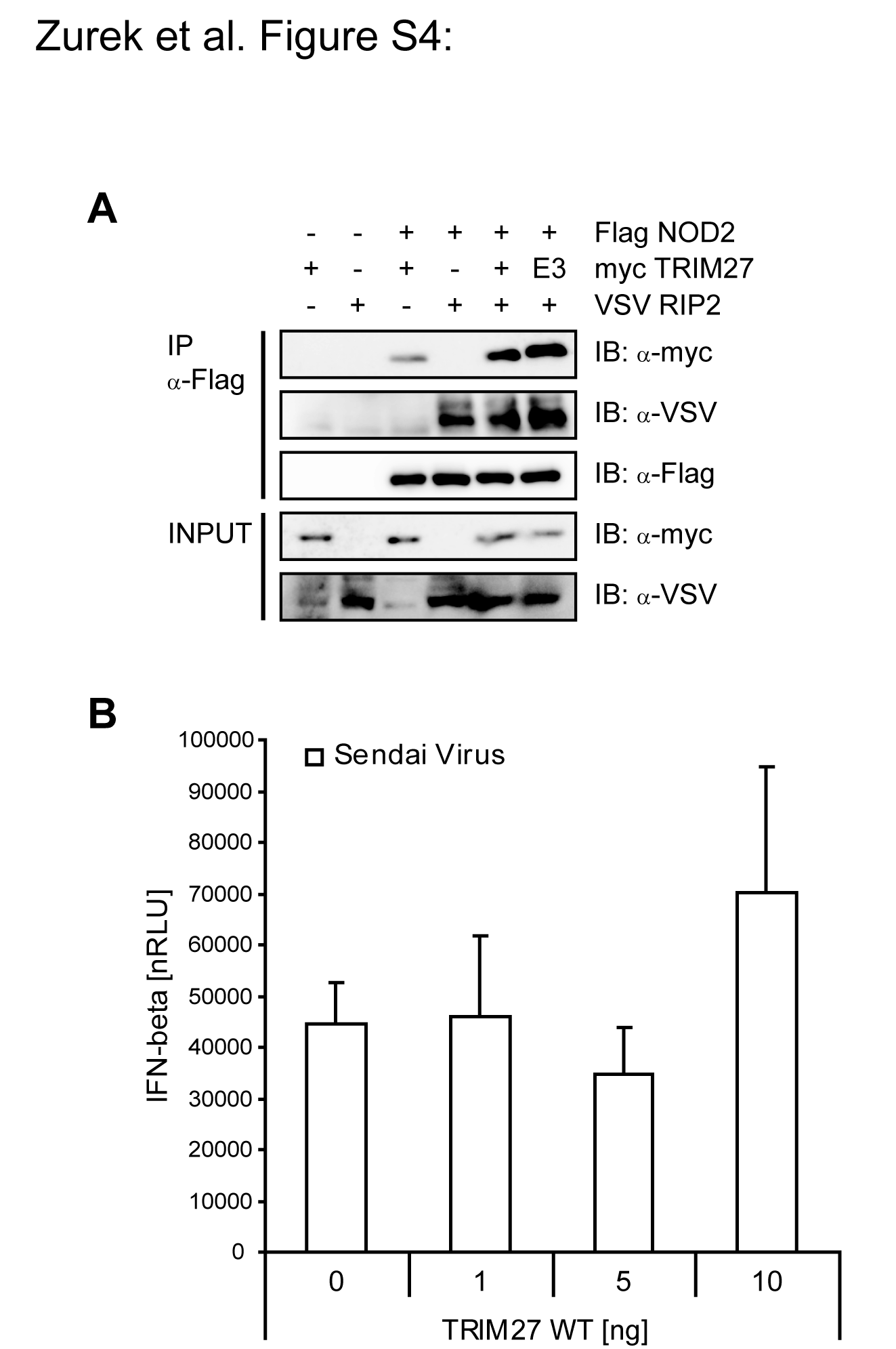

Supplement: Figure S4 — Effect of TRIM27 on RIP2/Nod2 interaction and IFN signalling. A. Lysates of HEK cells expressing the indicated proteins were subjected to immunoprecipitation using anti-Flag beads. Immunoblots of immunoprecipitates (IP) and total lysates (Input) were performed using the indicated antibodies. B. To determine the influence of TRIM27 on Sendai virus-induced IFN-β promoter activation, HEK293T cells were transfected with different amounts of TRIM27, as indicated, and an IFN-β promotor luciferase reporter system. Cells were then infected with 133 HAU/ml Sendai virus. Normalized luciferase activity (nRLU) is shown. Values are given as mean+SD. (TIF) [file pone.0041255.s004.tif]
